# Do All Stage IA Pancreatic Cancer Patients Need Adjuvant Chemotherapy?

**DOI:** 10.3390/cancers18081195

**Published:** 2026-04-08

**Authors:** John M. Lyons, Mei-Chin Hsieh, Kenneth C. Avanzino, Mohammad Al Efishat, Quyen Chu

**Affiliations:** 1 Department of Surgery, Louisiana State University Health Sciences Center, New Orleans, LA 70112, USA; 2 Our Lady of the Lake Cancer Center, Baton Rouge, LA 70808, USA; 3 Program of Epidemiology and Population Health, School of Public Health, LSUHSC, New Orleans, LA 70112, USA; 4 Department of Surgery, Howard University, Washington, DC 20059, USA; quyen.chu@howard.edu

**Keywords:** pancreas cancer, chemotherapy, stage IA, adjuvant therapy

## Abstract

Background: National guidelines recommend adjuvant chemotherapy (AC) following resection for all stages of pancreatic cancer (PDAC), but the benefit of AC in Stage IA disease remains unclear. The objective of this study was to identify a subgroup of patients with Stage IA PDAC that could possibly forego AC. Study Design: The National Cancer Database (NCDB) was queried to identify all patients with Stage IA PDAC diagnosed from 2010 to 2021. Patients who received AC were compared to those who did not. Multivariable analysis was conducted to identify risk factors associated with overall survival (OS). Results: There were 1421 patients eligible for analysis. On multivariable analysis, we found nine factors associated with worse overall OS: advanced age (*p* = 0.0414), lower median income (*p* = 0.0148), Medicare (*p* = 0.0180), higher-grade tumor histology (*p* = 0.0182), LVI (*p* = 0.0028), positive surgical margins (*p* = 0.0027), examination of fewer than 12 lymph nodes (*p* = 0.0395), and a length of stay greater than 7 days (*p* < 0.0001). OS was negatively correlated with an increased number of risk factors. Improved OS was observed following AC in patients with three (∆OS = +54 months; *p* = 0.0016) or four or more risk factors (∆OS = +11.4 months; *p* = 0.0250). However, patients with fewer than three risk factors did not experience improvement in OS following AC. Conclusions: AC does not appear to benefit Stage IA PDAC patients with fewer than three risk factors indicating that it may be safe to omit AC in these individuals.

## Simple Summary

National guidelines recommend chemotherapy for all patients with pancreatic cancer following surgery, but it is unclear if this benefits patients with early-stage disease. The purpose of this study was to investigate whether some patients with very early-stage pancreatic cancer could safely forgo chemotherapy following surgery. Multiple risk factors were found to be associated with worse overall survival such as advanced age, more aggressive tumor features, positive surgical margins, and prolonged hospital stay. Chemotherapy was associated with improved overall survival in those with multiple adverse clinicopathologic features but not in patients with fewer than three risk factors. The data from this retrospective study suggests that a subset of low-risk patients may experience limited benefit from chemotherapy.

## 1. Introduction

Pancreatic ductal adenocarcinoma (PDAC) remains an extremely lethal malignancy with five-year survival rates less than 15% [1]. Despite advancements in systemic therapy, even early-stage PDAC is associated with high recurrence rates [2]. Thus, adjuvant chemotherapy (AC) is currently recommended by the National Comprehensive Cancer Network (NCCN) and others for all stages of PDAC [3].

American Joint Committee on Cancer (AJCC) Stage IA PDAC includes tumors that measure ≤2 cm in greatest dimension and have no regional nodal involvement and no distant metastases [4]. Because of the aggressive nature of PDAC, stage IA disease is a rare diagnosis [5]. Although AC has improved OS in multiple randomized trials [6,7,8,9,10], early-stage patients are underrepresented in these trials which makes the benefit of AC in this subset of patients less clear.

Several retrospective studies have examined the effect of AC on survival in patients with Stage IA PDAC revealing mixed results. Ostapoff et al. analyzed 3909 patients with Stage IA or IB PDAC, using data from the National Cancer Database (NCDB) with AJCC 6th and 7th edition criteria [11]. In multivariable analysis, receipt of AC was associated with improved overall survival (OS) in both Stage IA and IB patients. Similarly, Turner et al. examined 1526 patients with Stage IA PDAC using a propensity score-matched analysis, and they also found that AC was significantly associated with improved OS [12]. Conversely, Izumo et al. analyzed 73 Stage IA PDAC patients treated in a single institution and found no association between AC and disease-specific survival (DSS) [13]. Zhang et al. conducted a dual-dataset analysis using cohorts from both Nanjing Medical University as well as the Surveillance, Epidemiology, and End Results (SEER) database [14]. Propensity score-matched analysis revealed no significant association between AC and OS in either dataset. Finally, Shaib et al. offered a more nuanced perspective. In their analysis of 876 patients with Stage IA PDAC, AC was associated with improved survival but only among those with tumors larger than 1 cm. No benefit from AC was observed in patients with tumors ≤1 cm [15]. These findings challenge the concept of universal adjuvant treatment for all Stage IA patients, and they suggest that a more tailored, individualized treatment approach may be warranted. It also implies that some patients with Stage IA PDAC may not require AC after surgery.

Preferred AC regimens for resected PDAC such as modified FOLFIRINOX [16,17] or gemcitabine-based regimens [10] carry the potential for adverse events—toxicities that are amplified in frail or elderly individuals. Additionally, unnecessary chemotherapy may diminish the effectiveness of subsequent systemic treatments by promoting the selection of chemoresistant clones [18]. Moreover, treatment for PDAC can impose significant financial toxicity, including high out-of-pocket costs, lost income, and persistent debt [19].

In this study, our aim was to investigate the clinical and pathological characteristics of patients with Stage IA PDAC and identify favorable risk factors predictive of positive outcomes. We hypothesized that we could identify a low-risk group of Stage IA patients for whom AC may offer limited benefit.

## 2. Methods

A participant user file (PUF) of the National Cancer Database (NCDB) was queried to identify all patients diagnosed with pathologic Stage IA PDAC from 2010 to 2021. We included all cases diagnosed in adult individuals using the following exocrine pancreatic adenocarcinoma histology codes: 8140, 8453, 8470, 8481, 8500, 8503.

Stage IA pancreatic cancer is defined similarly in both the AJCC 7th and 8th editions as a tumor confined to the pancreas that is 2 cm or smaller in greatest dimension (T1), with no regional lymph node involvement (N0) and no distant metastasis (M0). Analysis focused exclusively on patients with Stage IA (T1N0M0- tumor size less than 2 cm) pancreatic cancer diagnosed pathologically following upfront surgery. We excluded patients who experienced 30-day perioperative mortality, had neoadjuvant chemotherapy or radiation, or had histology other than adenocarcinoma.

Demographic and patient characteristics that were analyzed included age, sex, race/ethnicity, Charlson–Deyo Comorbidity Score, diagnosis year and treatment facility type. Additional factors included patient income, residential rural/urban status, distance from treatment facility, insurance status, and 30-day readmission rate. Tumor location within the pancreas was classified using the NCDB Primary Site variable (ICD-O-3 codes C25.0-C25.8) with locations within the gland being head (C25.0), body (C25.1), and tail (C25.2). Pancreatic tumors not coded in these specific anatomic locations were considered to have a location as “other.” Other tumor characteristics included histologic type, tumor size (<1 cm vs. 1–2 cm), grade, lymphovascular invasion (LVI), and number of lymph nodes examined.

The type of resection was determined by procedural codes and classified as either Whipple, Total pancreatectomy, or Pancreatectomy NOS. The post-surgical tumor margin status was reported as either no residual tumor, residual tumor, or unknown margin status. Length of stay after surgery was grouped as ≤7 days and >7 days. Patients listed as having any chemotherapy following surgery were defined as having adjuvant chemotherapy (AC). Overall survival was determined from date of diagnosis to the date of death from any cause or date of last follow up, and it was reported in months. For survival analysis, cases with missing or unknown values were excluded.

### Statistical Analysis

Descriptive statistics were used to summarize patients’ demographic characteristics, clinical features, and surgical-related factors, stratified by chemotherapy status. Pearson’s chi-square test was applied to examine unadjusted associations between categorical variables. Survival curves were estimated using the Kaplan–Meier method, and differences between groups were assessed with the log-rank test. To identify factors associated with all-cause mortality, both univariable and multivariable Cox proportional hazards regression models were fitted. Hazard ratios (HRs) with corresponding 95% confidence intervals (CIs) were reported. The proportional hazards assumption was evaluated using the Schoenfeld residuals test for primary exposure variable of interest. All statistical analyses were conducted using SAS version 9.4 (SAS Institute Inc., Cary, NC, USA). Statistical significance was determined using two-sided tests, with a *p*-value < 0.05 considered statistically significant.

## 3. Results

### 3.1. Demographics of the Entire Cohort

There were 1421 patients with Stage IA pancreatic cancer. The demographic characteristics of these patients are outlined in Table 1. The median age for the entire study group was 67 years old. The majority of patients were female (56%), and over two thirds of patients were white (79%). Most patients (54.6%) analyzed cohort had Medicare, lived in a metropolitan area (71.3%), and lived within 50 miles of the cancer reporting facility (65.9%). Fifty-three percent of patients received their treatment in an academic facility, and 63.7% of patients had 0 Charleson Deyo comorbidities (CCI) noted. The median tumor size was 1.5 cm, with most patients having a tumor in the pancreatic head (53%).

Although 83% of patients did not have LVI, 67% of patients had a tumor that was at least moderately differentiated. Surgical margins were negative in the majority of patients (93%). Median length of stay after surgery was 7 days, and only 9.4% of patients experienced a readmission within 30 days of discharge. The median OS for patients with Stage IA PDAC was 108 months.

### 3.2. Comparison of Patients Who Received AC Versus Those Who Did Not

Over half (56%) of the patients in this cohort received AC, and this proportion increased over time −52% received AC from 2010–2015 versus 59% from 2016–2022 (*p* = 0.0126). Patients who received AC were younger than those who did not (mean age 64.8 vs. 69.0; *p* < 0.0001). A higher proportion of patients in the AC group had private insurance (41.2% versus 28.1%; *p* < 0.0001), lived within 50 miles of the treatment facility (68.1% versus 63.1%; *p* = 0.0008) and had a comorbidity score of 0 (66.0% versus 60.7%; *p* = 0.0399). A higher proportion of patients in the AC group had tumors that were located in the pancreatic head (54.7% versus 52%; *p* = 0.0126), greater than 1 cm in size (75.9% versus 60%; *p* < 0.0001), and had LVI (11.9% versus 7.9%; *p* = 0.0336). Fewer patients in the AC group had well differentiated histology (20.1% versus 27.3%; *p* < 0.0001).

Patients in the AC group had a higher margin positivity rate (7.3% versus 3.5%; *p* = 0.0084) but they had a lower 30-day readmission rate (7.6% versus 11.7%; *p* = 0.0103). Additionally, a higher proportion of patients in the AC group had a hospital stay that was less than 7 days (62.9% versus 51.5%; *p* < 0.0001). Patients in the AC group experienced better OS (5-year survival 119 versus 88.9 months, *p* = 0.004) according to the log rank test.

### 3.3. Risk Factors Associated with Overall Survival

Following exclusions for missing or unknown variables, 961 patients were included in the survival analysis. In multivariate analysis of the entire cohort, AC was independently associated with improved OS (Table 2). This was observed in patients receiving both single-agent chemotherapy (HR: 0.697; 95% CI: 0.538–0.904; *p* = 0.0064) and multi-agent chemotherapy (HR: 0.587; 95% CI: 0.435–0.793; *p* = 0.005). Advanced age was also associated with worse OS, specifically among patients aged 50–64 (*p* = 0.0273) and those older than 75 years (*p* = 0.0414). Additional factors independently associated with worse overall survival included lower median income (*p* = 0.0148), Medicare insurance (*p* = 0.0180), higher-grade tumor histology (moderately differentiated, poorly differentiated, or undifferentiated; *p* = 0.0182), presence of LVI (*p* = 0.0028), positive surgical margins (*p* = 0.0027), examination of fewer than 12 lymph nodes (*p* = 0.0395), and a length of hospital stay greater than 7 days (*p* < 0.0001).

### 3.4. Survival Analysis According to Independent Risk Factors

**Figure 1:**
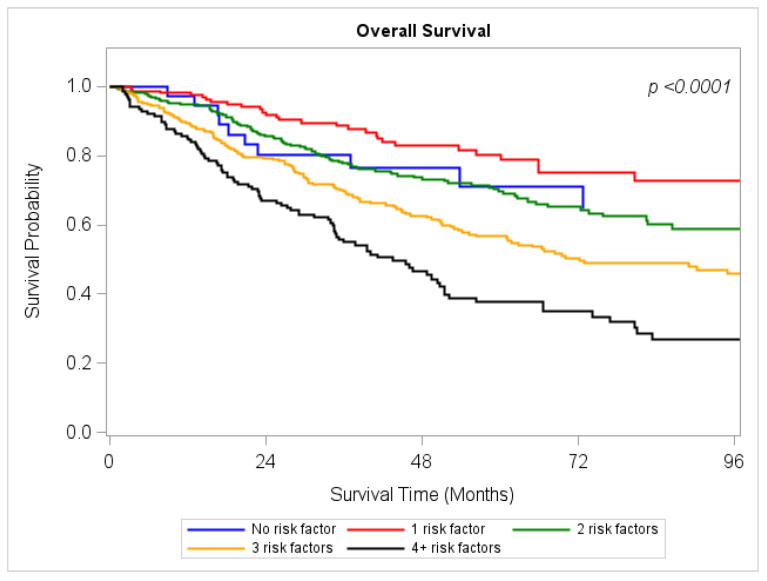
Kaplan–Meier Overall Survival Estimates of all patients with Stage IA PDAC according to Pathologic Risk Factors (*n* = 961) Risk factors analyzed were Tumor size > 1 cm, Moderate/poor differentiation, LVI, Greater than 12 lymph nodes examined, Positive surgical margins, LOS > 7 days.

The following six risk factors were further evaluated: tumor grade, LVI, surgical margins, lymph node harvest, and length of stay. There were 37 (3.9%) patients who had none (0%) of these risk factors. There were 158 (16.4%) patients who had one risk factor. There were 323 (33.6%) patients with two risk factors, 288 (30%) patients with three risk factors, and 155 (10.1%) patients with four or more risk factors. OS was analyzed according to the number of these risk factors patients harbored. These data are outlined in Table 3. Patients with 0 risk factors experienced a five-year survival rate of 92.9%, while those with 1, 2, 3, and 4 or greater risk factors experienced five-year survival rates of 78.8%, 69.0%, 56.5%, and 37.2% respectively. The corresponding survival curves are outlined in Figure 1.

**Figure 2:**
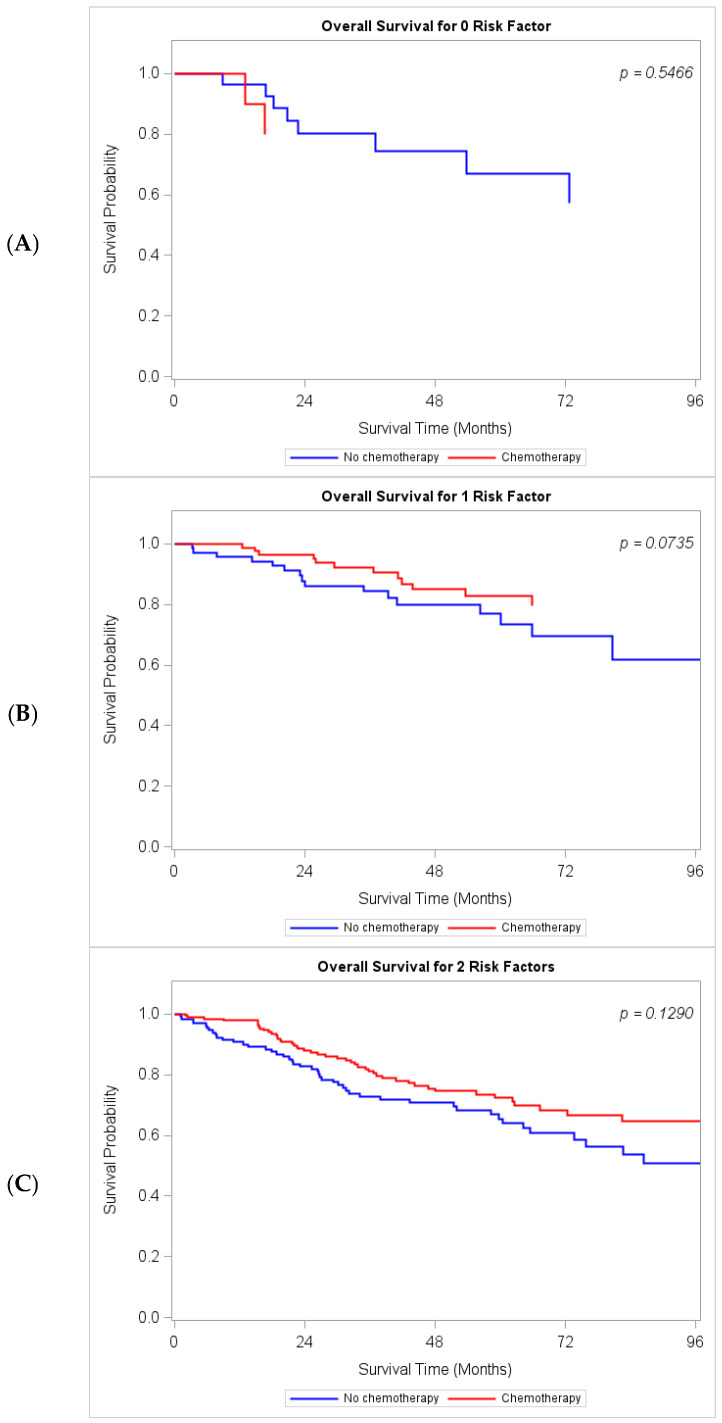

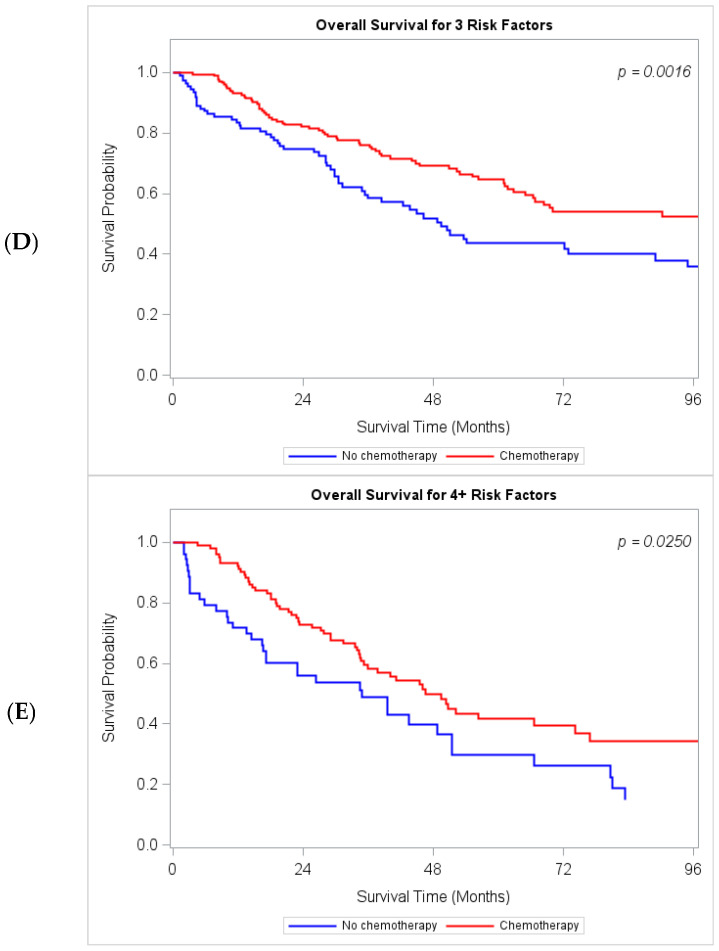
Kaplan–Meier Overall Survival estimates of patients who received and did not receive adjuvant chemotherapy (**A**) Patients with 0 Pathologic Risk Factors. (**B**) 1 Pathologic Risk Factor. (**C**) 2 Pathologic Risk Factors. (**D**) 3 Pathologic Risk Factors. (**E**) Greater than 3 Pathologic Risk Factors. Risk factors analyzed were Tumor size > 1 cm, Moderate/poor differentiation, LVI, Greater than 12 lymph nodes examined, Positive surgical margins, LOS > 7 days.

The median OS for patients with no risk factors was not reached. Among patients with no (0) risk factors, there was no significant difference in OS between those who received AC and those who did not (Figure 2). Similarly, no OS difference was observed following AC in patients with one or two risk factors. However, patients with three risk factors who received AC had significantly longer OS compared to those who did not (104.0 vs. 49.4 months; *p* = 0.0016). Improved OS was also observed in patients with four or more risk factors who received AC (46.6 vs. 34.9 months; *p* = 0.0250).

### 3.5. Consideration of Immortal Time Bias

We limited the study cohort to patients with Stage IA PDAC who underwent at least partial pancreatectomy, the recommended curative treatment for Stage I disease, thereby minimizing the potential for immortal time bias. To further evaluate this concern, a time-dependent Cox proportional hazards analysis was performed to model receipt of AC as a time-varying exposure. HRs from the time-dependent models are shown in Supplementary Table S1. The HR estimates were consistent over time for both single-agent and multi-agent regimens in unadjusted and adjusted analyses. The near-zero time interaction coefficients (β = 0.0016, *p* = 0.5547 in the unadjusted model; β = 0.0020, *p* = 0.4551 in the adjusted model) suggest no significant impact of survivor treatment selection bias on the observed associations.

## 4. Discussion

National guidelines recommend that all patients with PDAC receive AC following surgery regardless of stage [3]. We hypothesized that we could identify a low-risk group of early-stage PDAC patients who may derive limited benefit from AC. In order to test this, we studied over 1400 patients with AJCC Stage IA PDAC abstracted within the NCDB.

We found that AC was associated with improved OS compared with patients who did not receive AC (119 versus 88.9 months; *p* = 0.004). However, patients who received AC tended to be younger, had fewer comorbidities, lived closer to the treatment facility, and had a shorter hospital stay. Additionally, patients who received AC had more poorly differentiated tumors, higher incidence of LVI, higher margin positivity rate, and larger tumor size. Thus, these two comparison groups were very different sets of patients at baseline, and their differences imply a strong selection bias weakening the validity of a direct comparison. We therefore sought to identify independent risk factors that could allow for more meaningful patient stratification.

On multivariate analysis, we found nine risk factors independently associated with OS. Advanced age was independently associated with worse survival. Although some studies have found that younger patients with earlier stage PDAC have more aggressive grade [20], the worse OS of older patients is likely influenced by greater frailty and more comorbidities [21]. Having Medicare was also found to be independently associated with worse OS. This finding has been reported by many others and illustrates the profound influence that insurance has on the receipt of cancer care in the United States [22,23,24,25]. We found that higher median income was independently associated with improved OS. While many authors have used various definitions of and surrogates for income —including adjusted gross income [26], educational attainment [27], socioeconomic status [28], and area deprivation index—[29] lower income has been repeatedly linked to worse cancer outcomes.

Although these three risk factors—age, income, and insurance status—correlate with outcomes, they primarily reflect sociodemographic factors rather than tumor biology or quality of care. For that reason, they were intentionally excluded from the risk factor scoring both to reduce the possibility of introducing ageism into decision-making and to prevent structural bias against socially vulnerable individuals. Therefore, we based our stratification on the remaining six risk factors: tumor size > 1 cm, moderate and poor differentiation, LVI, examination of <12 lymph nodes, positive surgical margins, and length of stay >7 days.

By stratifying patients according to the number of independent prognostic factors they harbored, we found a clear gradient in OS, and we have identified a group that does not seem to benefit from AC. Patients with three or more risk factors experienced a more concerning prognosis, and survival among these individuals was improved with administration of AC. Thus, we believe these patients should be offered standard of care AC. On the other hand, those with fewer than three risk factors (outlined in Supplemental Table S2) had an excellent prognosis both with and without AC, and survival among these patients was not impacted by the administration of AC. These findings challenge the existing guidelines that recommend AC be administered to all stages of PDAC and support a more selective evaluation of patients with Stage IA disease [3]. However, they should be interpreted with caution and require prospective validation before influencing treatment decision.

The current study is a follow up to a previous NCDB analysis that also investigated the role of AC in Stage IA disease [12]. While the two studies have similarities, there are several important distinctions to highlight. First, the previous study obviously used an older dataset spanning 2006 to 2017 when single-agent chemotherapy was more prevalent. Secondly, only 46% of patients in the previous study received AC compared to 56% in the present analysis. Additionally, the previous study evaluated a slightly larger dataset due to the inclusion of additional histologic subtypes—such as tubular adenocarcinoma and mixed acinar-ductal adenocarcinoma—that were excluded from the current study. Although the previous study concluded that most patients with early-stage PDAC should receive chemotherapy, they did observe a subset of patients (*n* = 319) who did not appear to benefit from AC. We believe that the current study adds to the previous one by more clearly defining this group of lower-risk patients, i.e., those with fewer than three risk factors.

Other authors have also proposed stratification systems to select Stage I patients who should receive AC [30]. Zou et al. categorized patients into two groups according to pathologic risk factors—presence of perineural invasion and/or lymphovascular invasion—and found that only patients with “pathologic risk” experienced improved outcomes with AC. Furthermore, AC was an independent prognostic factor only among patients with pathological risk and not among those without pathological risk factors. Izumo et al. proposed a risk stratification system for patients with Stage IA PDAC using three factors: platelet-to-lymphocyte ratio ≥170, prognostic nutritional index <47.5, and pathological grade >1 [13]. They found that AC improved recurrence-free survival (RFS) only in high-risk patients, while those in the low-risk group observed no AC benefit. While both of these studies are single institution series limited by smaller sample sizes, their results align with those of the current study. Moreover, they further support the broader concept that AC does not benefit all patients with Stage I PDAC.

Although numerous emerging tools—such as proteomic profiling, circulating biomarkers, genetic signatures, liquid biopsy markers, and pancreatic stem cell analyses—hold great promise for more precise prognostication, their integration into routine clinical practice currently remains experimental in many circumstances [31]. Despite current trends favoring a neoadjuvant approach, we believe that studying the efficacy of adjuvant therapy in early-stage disease—where neoadjuvant approaches are less commonly applied—adds significant value.

The current study has limitations. A notable one is the inability to abstract the specific chemotherapy regimen or duration of treatment from the dataset. In addition, practice patterns regarding the recommendation of chemotherapy certainly evolved over time in response to newer publications, and this could not be controlled for in the current study. CA 19-9 is an important prognostic biomarker that has been incorporated into several existing scoring systems [32]. However, due to incomplete data for a significant number of patients, this variable could not be included in our analysis. Missing data may be related to institutional reporting variability and resource limitations potentially correlating to worse social determinants of health. This could further introduce selection bias into the analysis. Additionally, the behavior of some IPMN-associated malignancies is notably distinct, and our inclusion of IPMN-associated adenocarcinoma may be considered a study limitation [33]. However, because of the inability to reliably distinguish colloid and tubular subtypes in the dataset, and the fact that these cases comprised less than 20% of the entire cohort, we felt it reasonable to include them in this analysis, as have others [12]. Furthermore, in subset analysis, no statistically significant difference in OS was observed based on the presence or absence of an IPMN association. While this study is susceptible to immortal time bias, we accounted for this by remodeling the receipt of AC using a time-dependent Cox regression analysis (Supplemental Table S1). The near-zero time coefficient (β = 0.002; *p* = NS) implies that the analysis was not significantly impacted by survivor treatment selection bias. Finally, these findings are based on observational data accumulated from nonrandom treatment assignment, and they are inherently subject to selection bias and a degree of unmeasured confounding. Therefore, they should be carefully considered before being used to direct treatment decisions.

## 5. Conclusions

Among patients with resected Stage IA PDAC, AC was associated with improved OS in those with multiple adverse clinicopathologic features but not in patients with fewer than three risk factors. These data suggest that a subset of low-risk patients may experience limited benefit from adjuvant therapy; however, given the retrospective design and potential for residual confounding, these findings should be interpreted cautiously and require prospective validation prior to informing changes in standard clinical practice.

## Figures and Tables

**Table 1:** Baseline characteristics of patients with Stage IA psancreatic cancer *, 2010–2021.

Variables	All Patients (*n* = 1421)	No Chemotherapy (*n* = 615)	Adjuvant Chemotherapy (*n* = 806)	*p*-Value
	*n* (%)	*n* (%)	*n* (%)	
Age				<0.0001
18–49	96 (6.75%)	32 (5.2)	64 (7.9)	
50–64	460 (32.37%)	160 (26.0)	300 (37.2)	
65–74	487 (34.27%)	208 (33.8)	279 (34.6)	
75 and more	378 (26.59%)	215 (35.0)	163 (20.2)	
Sex				0.1314
Male	617 (43.42%)	281 (45.5)	336 (41.6)	
Female	804 (56.55%)	334 (54.3)	470 (58.3)	
Race/Ethnicity				0.595
White	1122 (78.96%)	496 (80.6)	626 (77.6)	
Black	137 (9.64%)	54 (8.7)	83 (10.2)	
Hispanic	83 (5.84%)	33 (5.3)	50 (6.2)	
Other	79 (5.56%)	32 (5.2)	47 (5.8)	
Insurance				<0.0001
Private Insurance	505 (35.52%)	173 (28.1)	332 (41.2)	
Medicaid	78 (5.49%)	38 (6.2)	40 (5.0)	
Medicare	777 (54.68%)	383 (62.3)	394 (48.9)	
Other Government	24 (1.69%)	7 (1.1)	17 (2.1)	
Uninsured/Unknown	37 (2.60%)	14 (2.3)	23 (2.9)	
Median income Quantiles 2012–2016				0.1953
<$40,227	192 (13.51%)	91 (14.7)	101 (12.5)	
$40,227–50,353	233 (16.39%)	112 (18.2)	121 (15.0)	
$50,354–63,332	278 (19.56%)	112 (18.2)	166 (20.6)	
≥$63,333	496 (34.91%)	213 (40.3)	283 (42.2)	
Unknown	222 (15.63%)	87 (14.1)	135 (16.7)	
Urban/Rural 2013				0.5504
Metro ≥ 250 K	1014 (71.31%)	431 (70.1)	583 (72.3)	
Urban ≥ 2500	315 (22.17%)	141 (22.9)	174 (21.2)	
Rural < 2500	24 (1.69%)	9 (1.4)	15 (1.8)	
Unknown	68 (4.78%)	34 (5.5)	34 (4.2)	
Distance from cancer reporting facility				0.0008
<50 miles	937 (65.94%)	388 (63.1)	549 (68.1)	
≥50 miles	272 (19.14%)	145 (23.6)	127 (15.8)	
Unknown	212 (14.92%)	82 (13.3)	130 (16.1)	
Facility type				0.5608
Academic	758 (53.33%)	338 (54.9)	420 (52.1)	
Non-academic	631 (44.39%)	264 (42.9)	367 (45.5)	
Unknown	32 (2.25%)	13 (2.1)	19 (2.3)	
Charlson-Deyo Score				0.0399
0	905 (63.68%)	373 (60.7)	532 (66.0)	
1	350 (24.63%)	154 (25.0)	196 (24.3)	
2	103 (7.25%)	53 (8.6)	50 (6.2)	
3+	63 (4.43%)	35 (5.7)	28 (3.5)	
Diagnosis Year				0.0126
2010–2015	494 (34.76%)	236 (38.4)	258 (32.0)	
2016–2021	927 (65.24%)	379 (61.6)	548 (68.0)	
Primary Site				0.0109
Head	761 (53.54%)	320 (52.0)	441 (54.7)	
Body	229 (16.12%)	88 (14.3)	141 (17.5)	
Tail	286 (20.13%)	127 (20.7)	159 (19.7)	
Other	145 (10.20%)	80 (13.0)	65 (8.1)	
Tumor size				<0.0001
<1 cm	440 (30.96%)	246 (40)	194 (24.1)	
1–2 cm	981 (69.04%)	369 (60)	612 (75.9)	
Median (cm)	2.9 (0.20%)	1.4	1.5	
Grade				<0.0001
Well differentiated	330 (23.23%)	168 (27.3)	162 (20.1)	
Mod. differentiated	722 (50.81%)	273 (44.4)	449 (55.7)	
Poorly differentiated	224 (15.76%)	75 (12.2)	149 (18.5)	
Undifferentiated	5 (0.35%)	2 (0.0)	3 (0.0)	
Unknown	140 (9.85%)	97 (15.7)	43 (5.3)	
LVI				0.0336
No	1182 (83.19%)	520 (84.5)	662 (82)	
Yes	145 (10.20%)	49 (7.9)	96 (11.9)	
Unknown	94 (6.62%)	46 (7.4)	48 (5.9)	
Number of lymph nodes examined				0.8351
<12	451 (31.72%)	197 (32.0)	254 (31.5)	
≥12	970 (68.26%)	418 (67.9)	552 (68.5)	
Resection Type				0.7344
Whipple	576 (40.53%)	254 (41.3)	322 (39.9)	
Total panc	160 (11.26%)	65 (10.5)	95 (11.7)	
Pancreatectomy, NOS	685 (48.22%)	296 (48.1)	389 (48.2)	
Surgical Margins				0.0084
No residual tumor	1333 (93.81%)	589 (95.7)	744 (92.3)	
With residual tumor	81 (5.70%)	22 (3.5)	59 (7.3)	
Unknown	7 (0.49%)	4 (0.0)	3 (0.0)	
LoS after surgery				<0.0001
≤7 days	824 (57.99%)	317 (51.5)	507 (62.9)	
>7 days	597 (42.01%)	298 (48.4)	299 (37.1)	
30 days readmission				0.0103
No	1287 (90.58%)	543 (88.3)	744 (92.3)	
Yes	134 (9.42%)	72 (11.7)	62 (7.6)	

* Pancreatic cancer was defined using the following histology codes: 8140 (adenocarcinoma, NOS), 8453 (invasive intraductal papillary mucinous carcinoma), 8470 (mucinous cystadenocarcinoma, NOS), 8481 (mucin-producing adenocarcinoma), 8500 (ductal carcinoma, NOS), and 8503 (intraductal papillary adenocarcinoma with invasion). Stage IA disease was defined according to both the 7th and 8th editions of the AJCC staging system as a tumor measuring ≤2 cm in greatest dimension (T1), with no regional lymph node involvement (N0) and no distant metastasis (M0).

**Table 2:** Multivariate analysis of overall survival among stage IA pancreatic cancer patients.

Variable	Hazard Ratio (95% CI)	*p*-Value
Chemotherapy		
No	1.000	
Single agent	0.697 (0.538–0.904)	0.0064
Multi-agent	0.587 (0.435–0.793)	0.0005
Age		
18–49	1.000	
50–64	2.217 (1.093–4.495)	0.0273
65–74	1.787 (0.842–3.797)	0.1308
75 and more	2.201 (1.031–4.699)	0.0414
Sex		
Male	1.000	
Female	1.098 (0.882–1.366)	0.4015
Race/Ethnicity		
Non-Hispanic white	1.000	
Non-Hispanic black	0.923 (0.623–1.367)	0.6882
Hispanic	0.832 (0.488–1.418)	0.4985
Other	1.272 (0.817–1.981)	0.2868
Insurance		
Private Insurance	1.000	
Medicaid	1.273 (0.767–2.112)	0.3496
Medicare	1.506 (1.073–2.114)	0.0180
Other Government	1.614 (0.705–3.693)	0.2570
Not Insured/Unknown	0.884 (0.438–1.784)	0.7302
Median income Quantiles 2012–2016		
<$40,227	1.000	
$40,227–50,353	0.819 (0.577–1.162)	0.2626
$50,354–63,332	0.928 (0.655–1.315)	0.6738
≥$63,333	0.660 (0.473–0.922)	0.0148
Urban/Rural 2013		
Metro ≥ 250 K	1.000	
Urban ≥ 2500	0.952 (0.707–1.28)	0.7426
Rural < 2500	0.79 (0.305–2.047)	0.6279
Distance from cancer reporting facility		
<50 miles	1.000	
≥50 miles	0.988 (0.723–1.352)	0.9420
Facility type		
Academic	1.000	
Non-academic	1.086 (0.871–1.355)	0.4613
Charlson-Deyo Score		
0	1.000	
1	0.969 (0.749–1.252)	0.8070
2	1.124 (0.763–1.655)	0.5554
3+	1.139 (0.640–2.028)	0.6575
Diagnosis year		
2010–2015	1.000	
2016–2021	0.840 (0.656–1.076)	0.1678
Primary Site		
Head	1.000	
Body	1.099 (0.790–1.530)	0.5756
Tail	1.010 (0.705–1.446)	0.9572
Other	1.115 (0.756–1.646)	0.5822
Tumor size		
<1 cm	1.000	
1–2 cm	1.648 (1.249–2.176)	0.0004
Grade		
Well differentiated	1.00	
Moderately/poorly/undifferentiated	1.361 (1.054–1.757)	0.0182
LVI		
No	1.000	
Yes	1.564 (1.167–2.096)	0.0028
Number of lymph nodes examined		
<12	1.000	
≥12	0.787 (0.627–0.989)	0.0395
Resection Type		
Whipple	1.000	
Total pancreatectomy	1.173 (0.825–1.667)	0.3750
Pancreatectomy, NOS	0.994 (0.755–1.308)	0.9638
Surgical Margins		
No residual tumor	1.000	
With residual tumor	1.804 (1.228–2.652)	0.0027
LoS after surgery		
≤7 days	1.000	
>7 days	1.620 (1.287–2.040)	<0.0001
30 days readmission		
No	1.000	
Yes	0.784 (0.544–1.129)	0.1911

**Table 3:** Overall survival of patients with Stage IA pancreatic cancer, 2010–2021.

Number of Risk Factors	All Patients		No Chemotherapy	Adjuvant Chemotherapy	*p*-Value
	*n* (%)	5-Years Survival (95% CI)	Median OS (95% CI)	Median OS (95% CI)	
0	37 (3.9)	92.9 (90.2–95.6)	NA	NA	0.5466
1	158 (16.4)	78.8 (69.7–85.4)	111 (80.6–NA)	NA	0.0735
2	323 (33.6)	69.0 (62.7–74.4)	127.6 (73.7–NA)	129.2 (106.5–NA)	0.129
3	288 (30.0)	56.5 (49.8–62.7)	49.4 (35.4–88.9)	104.1 (66.4–NA)	0.0016
≥4	155 (16.2)	37.2 (28.4–46.0)	34.9 (3.1–17.1)	46.6 (35.3–74.2)	0.025

Risk factors analyzed were Tumor size > 1 cm, Moderate/poor differentiation, LVI, Greater than 12 lymph nodes examined, Positive surgical margins, LOS > 7 days.

## Data Availability

The data presented in this study are available on request from the National Cancer Database Participant User Data File. Information on how to gain access and utilize such data was derived from the following resource: https://www.facs.org/quality-programs/cancer-programs/national-cancer-database/puf/ (accessed on 25 November 2024).

## Supplementary Materials

The following supporting information can be downloaded at: https://www.mdpi.com/article/10.3390/cancers18081195/s1, Supplement Table S1. Effect of adjuvant chemotherapy (AC) on survival among patients with stage IA pancreatic cancer at different follow-up periods using time-dependent Cox regression; Supplement Table S2. Characteristics of Stage IA PDAC patients with less than 3 risk factors, 2010–2021.

## Abbreviations

The following abbreviations are used in this manuscript:

PDAC	Pancreatic Ductal Adenocarcinoma
AC	Adjuvant Chemotherapy
NCBD	National Cancer Database
NCCN	National Comprehensive Cancer Network
AJCC	American Joint Committee in Cancer
OS	Overall Survival
LVI	Lymphovascular Invasion
