# Supplementary material for: Do All Stage IA Pancreatic Cancer Patients Need Adjuvant Chemotherapy?
Source: Cancers (Basel). 2026 Apr 8;18(8):1195. doi: 10.3390/cancers18081195 (PMC13115334; doi:10.3390/cancers18081195)
Supplement: Supplementary file 1 [file cancers-18-01195-s001.zip › cancers-4179884-supplementary.pdf]

Supplement Table S1. Effect of adjuvant chemotherapy (AC) on survival among patients with stage IA pancreatic cancer at different follow-up periods using time-dependent Cox regression

**Unadjusted HR and 95% CI**				
Coefficient of time-dependent AC			p-value	
0.00159			0.5547	
Effect of AC	HR	95% CI		p-value
Effect of single agent at t=6	0.7516	0.5666	0.997	0.0476
Effect of single agent at t=12	0.7588	0.5806	0.9917	0.0433
Effect of single agent at t=24	0.7734	0.6038	0.9906	0.0419
Effect of single agent at t=60	0.8188	0.6193	1.0825	0.1605
Effect of multi-agent at t=6	0.5495	0.3735	0.8085	0.0024
Effect of multi-agent at t=12	0.5548	0.3855	0.7983	0.0015
Effect of multi-agent at t=24	0.5654	0.4089	0.7819	0.0006
Effect of multi-agent at t=60	0.5986	0.4587	0.7812	0.0002
**Adjusted HR and 95% CI**				
Coefficient of time-dependent AC			p-value	
0.0020			0.4551	
Effect of AC	HR	95% CI		p-value
Effect of single agent at t=6	0.6804	0.5083	0.9106	0.0096
Effect of single agent at t=12	0.6886	0.5218	0.9088	0.0084
Effect of single agent at t=24	0.7053	0.5445	0.9136	0.0082
Effect of single agent at t=60	0.7579	0.5668	1.0136	0.0617
Effect of multi-agent at t=6	0.5385	0.3598	0.8059	0.0026
Effect of multi-agent at t=12	0.5450	0.3719	0.7986	0.0018
Effect of multi-agent at t=24	0.5582	0.3954	0.788	0.0009
Effect of multi-agent at t=60	0.5999	0.4481	0.803	0.0006

Adjusted for age, insurance, median income, diagnosis year, tumor size, grade, LVI, number of LN examined, margin, and length of stay after surgery

t is survival time measured in months

Supplement Table S2. Characteristics of Stage IA PDAC patients with less than 3 risk factors, 2010-2021

Variables	No chemotherapy (n=230)<br>n (%)	Adjuvant chemotherapy (n=288)<br>n (%)	P-value
Age			<0.0001
18-49	6 (25.0)	18 (75.0)	
50-64	68 (35.9)	121 (64.0)	
65-74	70 (42.9)	93 (57.0)	
75 and more	86 (60.5)	56 (39.4)	
Sex			0.8605
Male	104 (44.8)	128 (55.1)	

Female	126 (44.0)	160 (55.9)	
Race/Ethnicity			0.5891
Non-Hispanic white	193 (45.5)	231 (54.4)	
Non-Hispanic black	13 (34.2)	25 (65.7)	
Hispanic	12 (41.3)	17 (58.6)	
Other	12 (44.4)	15 (55.5)	
Insurance			0.0012
Private Insurance/Managed Care	63 (32.9)	128 (67.0)	
Medicaid	14 (51.8)	13 (48.1)	
Medicare	146 (51.7)	136 (48.2)	
Other Government	3 (37.5)	5 (62.5)	
Uninsured/Unknown	4 (40.0)	6 (60.0)	
Median income Quantiles 2012-2016			0.5374
< $40,227	33 (45.8)	39 (54.1)	
$40,227-50,353	52 (49.5)	53 (50.4)	
$50,354-63,332	50 (40)	75 (60)	
≥$63,333	95 (43.9)	121 (56.0)	
Urban/Rural 2013			0.8346
Metro ≥250K	166 (43.9)	212 (56.0)	
Urban ≥2500	61 (46.2)	71 (53.7)	
Rural <2500	3 (37.5)	5 (62.5)	
Distance from cancer reporting facility			0.0077
<50 miles	164 (41.2)	234 (58.7)	
≥50 miles	66 (55.0)	54 (45.0)	
Facility type			0.1830
Academic	138 (46.9)	156 (53.0)	
Non-academic	92 (41.0)	132 (58.9)	
Charlson-Deyo Score			0.4319
0	141 (43.12)	186 (56.8)	
1	59 (44.7)	73 (55.3)	
2	16 (44.4)	20 (55.5)	
3+	14 (60.8)	9 (39.1)	
Diagnosis Year			0.8032
2010-2015	64 (43.5)	83 (56.4)	
2016-2021	166 (44.7)	205 (55.2)	
Primary Site			0.0003
Head	105 (41.8)	146 (58.1)	
Body	35 (37.2)	59 (62.7)	
Tail	52 (43.3)	68 (56.6)	
Other	38 (71.7)	15 (28.3)	
Histology			0.0003
8140 Adenocarcinoma, NOS	107 (47.5)	118 (52.4)	
8453 Intraductal papillary mucinous neoplasm	51 (61.4)	32 (38.5)	
8470 Mucinous cystic neoplasm	2 (40.0)	3 (60.0)	

8481 Mucin-producing adenocarcinoma	68 (34.1)	131 (65.8)	
8500 Ductal adenocarcinoma	2 (33.3)	4 (66.6)	
8503 Intraductal papillary neoplasm	11 (61.1)	7 (38.8)	
Tumor size			<0.0001
<1cm	129 (54.8)	106 (45.1)	
1-2cm	101 (35.6)	182 (64.3)	
Median (cm)	1.4	1.5	
Grade			0.0449
Well differentiated	106 (50.2)	105 (49.7)	
Moderately differentiated	101 (41.5)	142 (58.4)	
Poorly differentiated	22 (34.9)	41 (65.0)	
Undifferentiated	1 (100.0)	0 (0.0)	
LVI			0.0191
No	230 (45.01)	281 (54.99)	
Yes	0 (0.00)	7 (100.00)	
Number of lymph nodes examined			0.6694
<12	35 (46.6)	40 (53.33)	
≥12	195 (44.0)	248 (55.98)	
Resection Type			0.4350
Wipple	86 (45.0)	105 (54.97)	
Total pancreatectomy	22 (36.6)	38 (63.33)	
Pancreatectomy, NOS	122 (45.6)	145 (54.31)	
Surgical Margins			0.7375
No residual tumor	227 (44.6)	282 (55.4)	
With residual tumor	3 (33.3)	6 (66.67)	
LoS after surgery			0.0003
≤7 days	158 (40.0)	237 (60.0)	
>7 days	72 (58.5)	51 (41.4)	
Median (days)	7	7	
30 days readmission			0.0577
No	206 (43.1)	271 (56.8)	
Yes	24 (58.5)	17 (41.4)	
